# Science in transition: open access to society?

**DOI:** 10.1007/s12471-013-0505-9

**Published:** 2013-12-24

**Authors:** E. E. van der Wall

**Affiliations:** Interuniversity Cardiology Institute of the Netherlands (ICIN) - Netherlands Heart Institute, Catherijnesingel 52, PO Box 19258, 3501 DG Utrecht, the Netherlands

In general, the current article-refereeing mechanism depends on trust in both the integrity of submissions and in peer reviewers. Nowadays, that trust is being tested by a disturbing change in scientific communication: *open access*. Unlike traditional journals, which rely largely on subscription revenue, many open-access publications earn their money through publication fees from authors. Profit is linked to volume, seemingly unlimited on the Internet. Although the open-access world includes many high-standard journals, abuse is prevalent, as an investigation by the journal *Science* recently established [1, 2]. To that purpose, *Science* journalist John Bohannon submitted fake papers with unmistakable scientific flaws to 304 open-access journals. It turned out that 157 of the journals had accepted the paper and 98 had rejected the paper. Of the remaining 49 journals, 29 seemed to be non-operational and 20 journals had yet to reply. Acceptance took 40 days on average, compared to 24 days for a rejection. Of the 255 papers that underwent the entire editing process to acceptance or rejection, about 60 % of the final decisions occurred with no sign of peer review. Of the 106 journals that evidently performed any review, 70 % ultimately accepted the paper. Most reviews focused exclusively on the paper’s layout, formatting, and language. Only 36 of the 304 submissions gave rise to review comments recognising any of the paper’s scientific problems, and 16 of those papers were accepted by the editors despite the poor reviews! To summarise, bogus research as concocted by *Science* can easily pass the threshold of publication in the open-access area; even 157 journals accepted the fake papers!

With open-access journals booming, debate is needed about how to ensure the credibility of scientific literature. Open-access pioneer Vitek Tracz believes that anonymous peer review is ‘*sick and collapsing under its own weight*’ [3]. As a remedy, Tracz has launched a new open-access journal in which reports—including all supporting data—are reviewed after they are posted online. As the number of published papers and the cost of doing research grows, there is an increasing need to predict *the societal impact*. The ability to publish papers and their underlying data in full on the Internet opens new possibilities to show true negative (or at least neutral) results. In the past, papers showing that a drug had no favourable effects failed to see the light of day. On the other hand, disseminating certain scientific information could pose a threat to safety and security. Scientists in industry, too, are struggling to define the limits of openness when communicating patented research, and whether some kinds of patents may actually suppress innovation [4].

How the dramatic shifts in scientific communication will affect the culture of research and processes for academic advancement and funding is far from clear. One thing becomes evident: science is becoming more of a public activity. Recently, this phenomenon has become a major issue in the Netherlands and has been coined as *Science in Transition* by five scientists/policymakers: Huub Dijstelbloem (Amsterdam), Frank Huisman (Utrecht), Frank Miedema (Utrecht), Jerry Ravetz (Oxford) and Wijnand Mijnhardt (Descartes Centre, Utrecht). These individuals voice a deeply-felt uncertainty and discontent on a number of aspects of the scientific system: the tools measuring scientific output, the publish-or-perish culture, the level of academic teaching, the scarcity of career opportunities for young scholars, the impact of science on policy, and the relationship between science, society and industry (www.scienceintransition.nl). Over the past years there has been an overemphasis on Hirsch indices and impact factors, even to such a point that these parameters became of crucial importance in awarding grants, career perspectives, and publication policies [5–7]. Obviously, this leads to gross manipulation of the scientific system affecting scientific integrity and inherent quality [8, 9]. Consequently, the checks and balances of the scientific system are in need of revision. To accomplish this, the above-mentioned group of *Science in Transition* advocate that science should be evaluated on the basis of its added value to society. The public should be given a better insight into the process of knowledge production: what parties play a role and what issues are at stake. Stakeholders from society should become more involved in this process, and have a bigger say in the allocation of research funding.

Although the desired ‘reformation’ of the scientific system might be refreshing and challenging, there are many questions to be answered. How does one measure the ‘added value to society’? How should one directly involve the public in the scientific process? Is scientific research useful when there is no direct clinical, practical, or societal impact [10]? When applied to the cardiovascular domain: is genetic research in the zebrafish to identify heart valve defects as useful as the development of a new bio-absorbable stent to better cure patients with an acute myocardial infarction? Which of these studies should be funded? Both studies or just the one with a clear public-appealing perspective? These are questions that should be answered by visionary scientists rather than by the general public. Only the former are truly capable of judging the potential impact to society with the aim to provide open access to the public.
